# Supplementary material for: Remote working and experiential wellbeing: A latent lifestyle perspective using UK time use survey before and during COVID-19
Source: PLoS One. 2024 Jul 25;19(7):e0305096. doi: 10.1371/journal.pone.0305096 (PMC11288641; doi:10.1371/journal.pone.0305096)
Supplement: S1 Appendix — (DOCX) [file pone.0305096.s001.docx]

| Survey | Number of Individuals | Percentage total: Individuals | Number of Activity-Location-Wellbeing Bundles | Percentage total: Bundles |
| --- | --- | --- | --- | --- |
| 2015 | 2,373 | 61.56 | 156,687 | 42.42 |
| 2016 | 291 | 7.55 | 41,777 | 11.31 |
| May-June 2020 | 323 | 8.38 | 46,333 | 12.54 |
| August 2020 | 370 | 9.60 | 53,054 | 14.36 |
| November 2020 | 498 | 12.92 | 71,543 | 19.37 |
