# Supplementary material for: Remote working and experiential wellbeing: A latent lifestyle perspective using UK time use survey before and during COVID-19
Source: PLoS One. 2024 Jul 25;19(7):e0305096. doi: 10.1371/journal.pone.0305096 (PMC11288641; doi:10.1371/journal.pone.0305096)
Supplement: S2 Appendix — (DOCX) [file pone.0305096.s002.docx]

| Time use categories | UKTUS 15 Activities | 4-Wave Activities |
| --- | --- | --- |
| Personal | Sleep | Sleep |
|  | Eating | Eating |
|  |  | Hygiene-related maintenance |
| Paid work | Primary employment | Primary employment |
|  | Secondary employment | Secondary employment |
|  | Formal education | Formal education |
| Non-paid work | Food management | Household chores |
|  | House upkeep | Child & elder care |
|  | Laundry | Volunteering |
|  | Gardening | Shopping, bank, misc. errands |
|  | Shopping |  |
|  | Childcare |  |
|  | Eldercare |  |
|  | Volunteer |  |
|  | Informal help |  |
|  | Religion |  |
| Leisure | Social life | Watching TV |
|  | Entertainment & culture | Reading |
|  | Physical exercise | Computer games |
|  | Arts | Eating out |
|  | Computing | Cinema, sport, theatre |
|  | Gaming | Time with friends and family |
|  | Reading | Telephone |
|  | TV | Recreational courses and other hobbies |
|  | Radio |  |
| Travel | Personal Travel | Travel by car |
|  | Work commute | Travel by cycle |
|  | Non-paid work travel | Travel by bus/tram/train/tube |
|  | Leisure travel | Travel by walking |
|  | Travel for other purposes | Travel others |
