# Supplementary material for: Remote working and experiential wellbeing: A latent lifestyle perspective using UK time use survey before and during COVID-19
Source: PLoS One. 2024 Jul 25;19(7):e0305096. doi: 10.1371/journal.pone.0305096 (PMC11288641; doi:10.1371/journal.pone.0305096)
Supplement: S4 Appendix — (DOCX) [file pone.0305096.s004.docx]

|  | Pre-COVID | | | During COVID | | |
| --- | --- | --- | --- | --- | --- | --- |
| Level of Enjoyment | Frequency | Percentage | Cumulative | Frequency | Percentage | Cumulative |
| 1 | 2,396 | 1.53% | 1.53% | 2,617 | 1.53% | 1.53% |
| 2 | 4,649 | 2.96% | 4.49% | 3,303 | 1.93% | 3.46% |
| 3 | 11,103 | 7.08% | 11.57% | 11,087 | 6.49% | 9.95% |
| 4 | 22,280 | 14.20% | 25.77% | 40,692 | 23.81% | 33.76% |
| 5 | 35,588 | 22.68% | 48.45% | 40,134 | 23.48% | 57.24% |
| 6 | 37,149 | 23.68% | 72.13% | 29,916 | 17.50% | 74.24% |
| 7 | 43,718 | 27.87% | 100.00% | 43,181 | 25.26% | 100.00% |
