# Supplementary material for: Remote working and experiential wellbeing: A latent lifestyle perspective using UK time use survey before and during COVID-19
Source: PLoS One. 2024 Jul 25;19(7):e0305096. doi: 10.1371/journal.pone.0305096 (PMC11288641; doi:10.1371/journal.pone.0305096)
Supplement: S5 Appendix — (DOCX) [file pone.0305096.s005.docx]

$$P_{jkt}= P_{t}^{X}P_{jt}^{AX}P_{kt}^{BX}$$

(Equation 1)

*X* is identifiable as a latent variable with $T=1\ldots t$ classes so that $P_{t}^{X}$is the probability of being in latent class *t*. $P_{jt}^{AX}$ is the probability of being in category *j* of variable A conditional on being in latent class *t*. $P_{kt}^{BX}$is the probability of being in category *k* of variable B conditional on being in latent class *t*. $P_{jkt}$ is the joint probability of being in category *j*, category *k,* and class *t.*

The responses of individual *i* on variables A and B are denoted each as $Y_{ijk}$, which is represented in the following section as a vector $Y_{i}$, such that the latent class model is defined as:

$$P\left( Y_{i} \right)=\sum_{t=1}^{T} P(X=t)P(Y_{i}|X=t)$$

(Equation 2)

Where $P(X=t)$ denote the class proportions. Applying a simple Bayesian formula to obtain the probability of belonging to latent class *t* given the observed response vector $Y_{i}$, the posterior membership probability is obtained from (Weller et al., 2020):

$$P\left( X=t | Y_{i} \right)=\frac{P(X=t)P(Y_{i}|X=t)}{P\left( Y_{i} \right)}$$

(Equation 3)

Further defining the assigned latent class membership of individual *i* as $W_{i}$*.* Adopting a proportional classification, which yields a soft partitioning with boundary *s* for assigning individuals to latent class *t* denoted as $W_{it}=P\left( W_{i}=t | Y_{i} \right)=P\left( X=t | Y_{i} \right).$ Allowing the classification error to be defined in terms of probability of the estimated value conditional on the true value:

$$P\left( W=s | X=t \right)=\frac{\sum_{i=1}^{N} P\left( X=t | Y_{i} \right)P\left( W=s | Y_{i} \right)}{P(X=t)}=\frac{\sum_{i=1}^{N} W_{it}W_{is}}{P(X=t)}$$

(Equation 4)

24 hourly categorical variables indicating activity type were used as the observed categorical variables in the models, together with categorical socioeconomic covariates including income quantile, education attainment, and occupation classification. Following the three-step model (Bolck et al., 2004), incorporating covariate with response vector $Z_{i}$ into the latent class model allows the covariates effects to be simultaneously estimated with the parameters defining the class-specific item distributions such that:

$$P\left( W=s|Z_{i} \right)=\sum_{t=1}^{T} P(X=t|Z_{i})P\left( W=s | X=t \right)$$

(Equation 5)

A multinomial logistical regression is used to parametrise $P(X=t|Z_{i})$. Using a maximum log-likelihood function, the model parameters above are estimated with:

$$LogL=\sum_{i=1}^{N} \log P\left( Y_{i} | Z_{i} \right)=\sum_{i=1}^{N} \log\sum_{t=1}^{T} P(X=t|Z_{i})P\left( W=s | X=t \right)$$

(Equation 6)

To ensure the global rather than local maximum is identified in the maximum likelihood estimation, Newton-Raphson stepping mechanism guarantees convergence if the initial estimate $x_{0}$ are close enough to the true estimates (Smith, 1998), as shown in the formula:

$$x_{n+1}=x_{n}-\frac{f(x_{n})}{f'(x_{n})}$$

(Equation 7)

To narrow the initial estimates to aid model convergence under Newton method, we apply a two-step integration process in the generalised linear mixed model used for LCA: Laplacian approximation was used in the first round of integration, the resulting matrix was then used as the starting value for the more accurate mean-variance adaptive Gauss-Hermite quadrature integration method (Rabe-Hesketh et al., 2002):

$$\int_{-\infty}^{\infty} L(\beta_{0}, \beta_{1};Y_{i}|X=t)P(X=t|Z_{i})dx$$

(Equation 8)

$$LogL\left( \beta_{0}, \beta_{1};Y_{i} | X=t \right)=Y_{i}\left( \beta_{0}+\beta_{1}x \right)-\log(1+e^{\beta_{0}+\beta_{1}x})$$

(Equation 9)

One to six class models are iteratively attempted, the final class size within each working mode was determined using the Akaike's information criterion (AIC) and Bayesian information criterion (BIC) values, average latent class probabilities, and substantive interpretation of the classes identified:

$$AIC=-2\log L\left( \hat{\theta} \right)+2k$$

(Equation 10)

$$BIC=-2\log L\left( \hat{\theta} \right)+k\log n$$

(Equation 11)

Where $\theta$ is the vector of model parameters and $k$ is the number of estimated parameters. $L\left( \hat{\theta} \right)$ is the candidate model when evaluated at the maximum likelihood estimate of $\theta$. LCA was conducted using pooled samples from both pre- and during COVID-19. After LCA, the samples were then separated into pre- and during COVID-19 groups according to the survey date.
