# Supplementary material for: Remote working and experiential wellbeing: A latent lifestyle perspective using UK time use survey before and during COVID-19
Source: PLoS One. 2024 Jul 25;19(7):e0305096. doi: 10.1371/journal.pone.0305096 (PMC11288641; doi:10.1371/journal.pone.0305096)
Supplement: S6 Appendix — (DOCX) [file pone.0305096.s006.docx]

| Working Mode | Latent class size | AIC | BIC |
| --- | --- | --- | --- |
| Homeworker | 1 | 41961.45 | 42430.36 |
|  | 2 | 38903.11 | 39782.32 |
|  | 3 | 38060.85 | 39316.16 |
|  | 4 | 38624.38 | 39682.53 |
|  | 5 | 38847.03 | 39873.82 |
|  | 6 | 39873.13 | 40341.84 |
| Commuter | 1 | 108888.00 | 109442.80 |
|  | 2 | 99457.26 | 100555.3 |
|  | 3 | 97296.77 | 98920.68 |
|  | 4 | 95493.31 | 97591.10 |
|  | 5 | 95208.19 | 97814.54 |
|  | 6 | 96445.05 | 98403.92 |
| Hybrid | 1 | 22629.53 | 23019.23 |
|  | 2 | 21302.53 | 22056.79 |
|  | 3 | 20911.47 | 21959.05 |
|  | 4 | 20990.47 | 22038.80 |
|  | 5 | 21330.45 | 22897.63 |
|  | 6 | Non-convergent | Non-convergent |
