# Supplementary material for: Remote working and experiential wellbeing: A latent lifestyle perspective using UK time use survey before and during COVID-19
Source: PLoS One. 2024 Jul 25;19(7):e0305096. doi: 10.1371/journal.pone.0305096 (PMC11288641; doi:10.1371/journal.pone.0305096)
Supplement: S7 Appendix — (DOCX) [file pone.0305096.s007.docx]

$$H=-\sum_{i=1}^{N} X_{i}\log_{2} X_{i}$$

Shannon entropy (H) is a measure of the uncertainty of the value taken on by our variable investigated. $X_{i}$ denotes the probability that the variable X would take on the value observe at $X_{i}$.

In the case of spatial flexibility, there are three possible workplaces: home, office, other. $X_{i}$ is the probability density function of the three workplaces over the working day for all individuals belonging to the same lifestyle. Shannon entropy is calculated for each lifestyle and normalised using the min-max feature scaling formula.

For temporal flexibility, there are also three possible activities: personal, leisure, other. $X_{i}$ is the probability density function of enjoyable breaks over the working day for all individuals belonging to the same lifestyle. Shannon entropy is calculated for each lifestyle and normalised using the min-max feature scaling formula.

$$H^{'}= \frac{H-H_{min}}{H_{max}-H_{min}}$$

Where $H^{'}$ is the normalised entropy that has a range of 0-1, where 0 represents the highest level of synchronicity and 1 represents highest level of flexibility in relativity to the latent lifestyles.
