# Supplementary material for: Remote working and experiential wellbeing: A latent lifestyle perspective using UK time use survey before and during COVID-19
Source: PLoS One. 2024 Jul 25;19(7):e0305096. doi: 10.1371/journal.pone.0305096 (PMC11288641; doi:10.1371/journal.pone.0305096)
Supplement: S9 Appendix — (DOCX) [file pone.0305096.s009.docx]

|  | | Before COVID-19 | | During COVID-19 | |
| --- | --- | --- | --- | --- | --- |
|  |  | Coef | P>z | Coef | P>z |
| Time Category | Personal | 0.54 | 0.00 | 0.98 | 0.00 |
|  | Non-paid Work | 0.34 | 0.00 | 1.03 | 0.00 |
|  | Leisure | 1.23 | 0.00 | 1.34 | 0.00 |
|  | Travel | -0.05 | 0.26 | 0.55 | 0.00 |
| Lifestyle | Emergent Homeworker (EH) | 0.21 | 0.02 | -0.15 | 0.00 |
|  | Highflying Homeworker (HH) | 0.84 | 0.00 | 0.12 | 0.01 |
|  | Domestic Home-worker (DH) | 0.26 | 0.05 | -0.12 | 0.11 |
|  | Long-distance Commuter (LC) | -0.24 | 0.00 | -0.17 | 0.00 |
|  | Half-day Commuter (HC) | 0.33 | 0.00 | 0.19 | 0.00 |
|  | Nightshift Commuter (NC) | 0.22 | 0.09 | -0.37 | 0.04 |
|  | Conventional Hybrid (CH) | -0.09 | 0.14 | 0.34 | 0.00 |
|  | Long-hour Hybrid (LH) | 0.16 | 0.10 | 0.39 | 0.00 |
|  | Mobile Hybrid (MH) | 0.15 | 0.39 | -0.39 | 0.00 |
| Sex | Female | 0.05 | 0.01 | -0.23 | 0.00 |
| Age Group | 25-34 | -0.15 | 0.00 | 0.13 | 0.00 |
|  | 35-44 | -0.04 | 0.34 | 0.01 | 0.83 |
|  | 45-54 | -0.14 | 0.00 | -0.18 | 0.00 |
|  | 55-64 | 0.03 | 0.49 | -0.18 | 0.00 |
| Education | GCSEs | -0.03 | 0.57 | 2.11 | 0.00 |
|  | A Levels | -0.17 | 0.00 | 2.30 | 0.00 |
|  | First degree/ undergrad | -0.18 | 0.00 | 1.96 | 0.00 |
|  | Higher degree | -0.36 | 0.00 | 2.09 | 0.00 |
| Occupation | Skilled manual worker | -0.03 | 0.27 | 0.29 | 0.00 |
|  | Clerical / administrative | -0.11 | 0.01 | -0.19 | 0.00 |
|  | Supervisory/ junior managerial | -0.09 | 0.02 | -0.08 | 0.03 |
|  | Intermediate managerial | -0.09 | 0.01 | -0.10 | 0.01 |
|  | Higher managerial | -0.27 | 0.00 | -0.02 | 0.67 |
| Income | £1,000-1,999 | 0.27 | 0.00 | -0.53 | 0.00 |
|  | £2,000-2,999 | 0.12 | 0.00 | -0.40 | 0.00 |
|  | £3,000-3,999 | 0.02 | 0.54 | -0.55 | 0.00 |
|  | £4,000-5,000 | -0.18 | 0.00 | -0.60 | 0.00 |
|  | More than £5,000 | -0.13 | 0.00 | -0.26 | 0.00 |
| Marital Status | Married/living with partner | 0.16 | 0.00 | 0.30 | 0.00 |
|  | Divorced/widowed | 0.17 | 0.00 | 0.11 | 0.00 |
| Region | Yorkshire & Humberside | 0.12 | 0.01 | -0.32 | 0.00 |
|  | East Midlands | 0.05 | 0.23 | -0.75 | 0.00 |
|  | East Anglia | 0.17 | 0.00 | -0.25 | 0.00 |
|  | South East | -0.08 | 0.04 | -0.51 | 0.00 |
|  | South West | 0.07 | 0.14 | -0.76 | 0.00 |
|  | West Midlands | 0.26 | 0.00 | -0.62 | 0.00 |
|  | North West | 0.12 | 0.01 | -0.17 | 0.00 |
|  | Scotland | 0.48 | 0.00 | -1.36 | 0.00 |
|  | Wales | 0.07 | 0.21 | -0.49 | 0.00 |
|  | Northern Ireland | 0.44 | 0.00 | -0.39 | 0.00 |
|  | North East | 0.53 | 0.00 | -0.34 | 0.00 |
| TimeCategory#Lifestyle | Personal # EH | 0.18 | 0.18 | -0.16 | 0.01 |
|  | Personal # HH | -0.69 | 0.00 | -0.40 | 0.00 |
|  | Personal # DH | 0.06 | 0.72 | 0.18 | 0.06 |
|  | Personal # LC | -0.02 | 0.85 | -0.19 | 0.06 |
|  | Personal # HC | -0.13 | 0.12 | -0.21 | 0.05 |
|  | Personal # NC | 0.33 | 0.02 | 0.40 | 0.04 |
|  | Personal # CH | 0.03 | 0.76 | -0.44 | 0.00 |
|  | Personal # LH | -0.33 | 0.05 | -0.22 | 0.09 |
|  | Personal # MH | 0.08 | 0.71 | -0.24 | 0.10 |
|  | NPW # EH | -0.07 | 0.62 | -0.56 | 0.00 |
|  | NPW # HH | -0.68 | 0.01 | -2.24 | 0.00 |
|  | NPW # DH | -0.29 | 0.06 | -0.73 | 0.00 |
|  | NPW # LC | -0.11 | 0.32 | -0.36 | 0.09 |
|  | NPW # HC | -0.23 | 0.01 | 0.74 | 0.00 |
|  | NPW # NC | 0.04 | 0.79 | 0.15 | 0.48 |
|  | NPW # CH | 0.07 | 0.55 | -0.61 | 0.00 |
|  | NPW # LH | 0.24 | 0.29 | -0.50 | 0.22 |
|  | NPW # MH | -0.19 | 0.35 | -0.26 | 0.17 |
|  | Leisure # EH | 0.06 | 0.73 | -0.29 | 0.00 |
|  | Leisure # HH | -1.83 | 0.00 | -0.37 | 0.02 |
|  | Leisure # DH | -0.37 | 0.03 | -0.24 | 0.04 |
|  | Leisure # LC | -0.11 | 0.38 | -1.11 | 0.00 |
|  | Leisure # HC | -0.29 | 0.01 | -0.22 | 0.10 |
|  | Leisure # NC | 0.04 | 0.79 | 0.34 | 0.10 |
|  | Leisure # CH | -0.11 | 0.45 | -0.50 | 0.00 |
|  | Leisure # LH | 0.28 | 0.42 | -0.81 | 0.00 |
|  | Leisure # MH | -0.16 | 0.47 | -0.09 | 0.59 |
|  | Transport # EH | -0.51 | 0.01 | 0.05 | 0.66 |
|  | Transport # HH | -0.35 | 0.35 | 0.39 | 0.04 |
|  | Transport # DH | 0.01 | 0.97 | 0.01 | 0.93 |
|  | Transport # LC | 0.15 | 0.12 | 0.58 | 0.00 |
|  | Transport # HC | -0.03 | 0.74 | 0.08 | 0.61 |
|  | Transport # NC | 0.32 | 0.04 | 0.48 | 0.03 |
|  | Transport # CH | 0.07 | 0.53 | -0.02 | 0.89 |
|  | Transport # LH | -0.03 | 0.88 | 0.86 | 0.00 |
|  | Transport # MH | -0.08 | 0.73 | -0.11 | 0.57 |
