# Supplementary material for: Remote working and experiential wellbeing: A latent lifestyle perspective using UK time use survey before and during COVID-19
Source: PLoS One. 2024 Jul 25;19(7):e0305096. doi: 10.1371/journal.pone.0305096 (PMC11288641; doi:10.1371/journal.pone.0305096)
Supplement: Attachment — Submitted filename: Response to Reviewers.docx [file pone.0305096.s010.docx]

**Responses to Reviewers**

We would like to thank the Reviewer for taking time to review our manuscript and for the constructive comments provided, which help us improve the quality of the paper. Please find below our point-to-point responses.

**Responses to the Editor**

1. I am particularly concern with methods, in addition that the paper needs clarity and the inclusion of recent economic literature on remote work. Authors use two cross-sections of UKTUS and then they can derive correlations. Consequently, the redaction should be appropriate in this context (e.g. avoiding causal terms as "impact").

Response: Thank you for the cogent comments. We confirm that the study is focused primarily on correlations. We have thus refined our wording to avoid alluding to causality in the manuscript. We also added the below paragraph on recent economic literature related to remote work:

*“A systematic review on the impact of remote work on productivity found stark contrast where before the pandemic 79% studies showed positive effects of remote work, that figure dropped to 23% during the pandemic (Hackney et al., 2022). Preliminary evidence show that remote workers experience more anxiety around job security (Fosslien & Gottlieb-Cohen, 2023), and are more prone to loneliness (Miyake et al., 2022). Cell-phone-based mobility data reveal trips to the CBD stabilising at 60% of pre-pandemic levels, suggesting remote workers will make up a significant portion of the future workforce (Monte et al., 2023).”*

2. In your Data Availability statement, you have not specified where the minimal data set underlying the results described in your manuscript can be found. PLOS defines a study's minimal data set as the underlying data used to reach the conclusions drawn in the manuscript and any additional data required to replicate the reported study findings in their entirety. All PLOS journals require that the minimal data set be made fully available. For more information about our data policy, please see [http://journals.plos.org/plosone/s/data-availability](https://eur03.safelinks.protection.outlook.com/?url=http%3A%2F%2Fjournals.plos.org%2Fplosone%2Fs%2Fdata-availability&data=05%7C01%7Cjc2205%40cam.ac.uk%7Cad754d1892494b5ed9a008db7166e11f%7C49a50445bdfa4b79ade3547b4f3986e9%7C1%7C0%7C638228458894602075%7CUnknown%7CTWFpbGZsb3d8eyJWIjoiMC4wLjAwMDAiLCJQIjoiV2luMzIiLCJBTiI6Ik1haWwiLCJXVCI6Mn0%3D%7C3000%7C%7C%7C&sdata=iQ1%2BMN2yanHutv7m2e6purgESp6E%2B1u3UH%2FfQNTXMIs%3D&reserved=0).

Upon re-submitting your revised manuscript, please upload your study’s minimal underlying data set as either Supporting Information files or to a stable, public repository and include the relevant URLs, DOIs, or accession numbers within your revised cover letter. For a list of acceptable repositories, please see [http://journals.plos.org/plosone/s/data-availability#loc-recommended-repositories](https://eur03.safelinks.protection.outlook.com/?url=http%3A%2F%2Fjournals.plos.org%2Fplosone%2Fs%2Fdata-availability%23loc-recommended-repositories&data=05%7C01%7Cjc2205%40cam.ac.uk%7Cad754d1892494b5ed9a008db7166e11f%7C49a50445bdfa4b79ade3547b4f3986e9%7C1%7C0%7C638228458894602075%7CUnknown%7CTWFpbGZsb3d8eyJWIjoiMC4wLjAwMDAiLCJQIjoiV2luMzIiLCJBTiI6Ik1haWwiLCJXVCI6Mn0%3D%7C3000%7C%7C%7C&sdata=O1BSSSrEhI7YX%2FrLJtUCMzZgPk6NXO41TqZ8d4OG4G8%3D&reserved=0). Any potentially identifying patient information must be fully anonymized.

Response: Our data is openly available through the UK Data Service. The DOIs are included under the “Data repository” section. The STATA codes are available via our GitHub link, also included under the “Code availability” section of the manuscript.

3.  Please include captions for your Supporting Information files at the end of your manuscript, and update any in-text citations to match accordingly. Please see our Supporting Information guidelines for more information: [http://journals.plos.org/plosone/s/supporting-information](https://eur03.safelinks.protection.outlook.com/?url=http%3A%2F%2Fjournals.plos.org%2Fplosone%2Fs%2Fsupporting-information&data=05%7C01%7Cjc2205%40cam.ac.uk%7Cad754d1892494b5ed9a008db7166e11f%7C49a50445bdfa4b79ade3547b4f3986e9%7C1%7C0%7C638228458894602075%7CUnknown%7CTWFpbGZsb3d8eyJWIjoiMC4wLjAwMDAiLCJQIjoiV2luMzIiLCJBTiI6Ik1haWwiLCJXVCI6Mn0%3D%7C3000%7C%7C%7C&sdata=o50pgMF6b8wn331mmr8d%2FdxC5erS8Sw3NtmsA29HzVs%3D&reserved=0).

Response: Thank you for outlining the additional requirements. The supporting information files and the in-text citations have been adjusted to the journal guidelines.

**Responses to Reviewer #1**
This paper studies the relationship between experiential well-being (ExWB) and remote work, while controlling for other relevant factors, such as hours of work, occupation, and family-related variables, such as marital status. Using time surveys from the UK, before and during the COVID-19 pandemic, the authors employ latent class models to examine the heterogeneous impacts of ExWB across distinct activity types, time of day, and lifestyles.

Overall, the paper is well-written and presents interesting findings. However, there are several concerns regarding the study's contribution and the validity of the results. These concerns may require further clarification and elaboration in order to strengthen the study’s contribution and robustness.

Concerns

1. The abstract is quite long. The authors should focus on the most important finding(s) of their paper.

Response: Thank you for this cogent suggestion. The abstract has been reduced from 279 words to 207 words. The updated abstract is quoted here for your reference:

*“Mental health in the UK had deteriorated compared with pre-pandemic trends. Existing studies on heterogenous wellbeing impacts of COVID-19 tend to segment population based on isolated socio-economic and demographic indicators, notably gender, income and ethnicity, while a more holistic and contextual understanding of such heterogeneity among the workforce seems lacking. This study addresses this gap by 1) combining UK time use surveys collected before and during COVID-19, 2) identifying latent lifestyles within three working mode groups (commuter, homeworker and hybrid worker) using latent class model, and 3) quantifying nuanced experiential wellbeing (ExWB) impacts across workers of distinct lifestyles. It was found that the direction and magnitude of ExWB impact were not uniform across activity types, time of day, and lifestyles. The direction of ExWB impact during usual working hours (6am-6pm) varied in accordance with lifestyle classifications. Specifically, ExWB decreased for all homeworkers but increased significantly for certain hybrid workers. Magnitude of ExWB impact correlated strongly with lifestyle. To understand the significant heterogeneity in ExWB outcomes, a spatial-temporal conceptualisation of working flexibility is developed to explicate the strong yet complex correlations between wellbeing and lifestyles. The implications to post-pandemic “back-to-work” policies are 1) continued expansion of hybrid working optionality, 2) provide wider support for lifestyle adaptation and transitions.”*

2. Authors examine the relationship between experiential well-being (ExWB) and remote work, including in their analysis other factors, such as employment characteristics, i.e., hours of work, occupation, or family factors, such as marital status. There is literature that relates parental status (motherhood/parenthood) to remote work and well-being. Authors should justify why they haven’t included in their analysis any indicators for parental status, for example number of children.

Response: Thank you for this important point regarding the lack of ‘parental status’ in our model. While we agree parental status is an important indicator of sociodemographic characteristics, there are several concerns that led us to exclude it from our study. Firstly, there is a significant non-response issue in questions relating to number of children. For instance, in the during-COVID data set there are 227 individuals (out of a total 1,191 samples) who provided no information on whether or not they have children in their household. Excluding these samples would reduce the sample size by roughly 20%, which is likely to lead to identification issues in the model. Moreover, as both childcare and eldercare hours are highly endogenous to the enjoyment of non-paid work, we believe the inclusion of parental status appears outside the scope of this paper. That said, we are interested in looking more closely at the non-paid work activities and analyse the specific impacts of child as well as elderly care in our future workstream. We added the following to the Limitations section of the manuscript to clarify on this point:

*“The logistical regression model does not include parental status and household composition as covariates out of concern for endogeneity. While household composition (including parental status) is an important indicator of sociodemographic characteristic, childcare and eldercare hours are highly endogenous to the enjoyment of non-paid work. The relationship between care obligations and ExWB warrant further study before we can address the potential endogeneity. Furthermore, there is a significant non-response issue in questions relating to number of children, affecting 20% of study samples. Inclusion of the covariate would significantly reduce the samples size, likely to lead to identification issues. We believe the inclusion of parental status alone would be problematic and more research is required to differentiate and control for these care obligations.”*

3. “The direction and magnitude of pandemic impact on ExWB were heterogenous across time of the day, activity types and lifestyles”. Authors acknowledge the heterogeneous effects of socio-economic factors on ExWB; they should explain better how latent class models help to deal with these heterogeneous effects in the present study.

Response: Thank you for raising the point on heterogenous ExWB changes. The use of latent class models is novel and effective in the sense that it enables a holistic classification of worker, which could hardly be achieved by using conventional, single socio-economic variables. Our empirical analysis showed that the direction and magnitude of wellbeing changes correlate strongly with the lifestyles endogenously identified from the latent class models. We agree with the reviewer that there is untapped potential to use the latent lifestyles as a unified covariate to better control for heterogeneity in the workforce, which is an exciting future workstream for us.

4. I have concerns about how novel is the use of latent class models and time-use survey data. For example:
Lee, Y. Activity Profiles among Older Adults: Latent Class Analysis Using the Korean Time Use Survey. Int. J. Environ. Res. Public Health 2021, 18, 8786. <https://doi.org/10.3390/ijerph18168786>, uses latent class models along with time-use survey data in a very similar way. Authors should acknowledge relevant to the use of latent class models literature and time-use survey data and motivate better their own contribution.

Response: Thank you for mentioning this relevant paper. We have added a paragraph of detailed review on this paper into our literature review section. From the title and abstract of Lee (2021), the latent class analysis application to time use survey does seem to be a methodological overlap. However, our paper makes several distinct and novel contributions, thus differ from Lee’s methodology in a significant way.

Firstly, Lee’s paper does not consider the timing (hence the sequence) of activities in the latent class assignment. Our latent classes represent people who engage in similar activities around the similar times of day, as illustrated in our tempogram in Figure 1. Distinguishing the timing and sequence of activities is thus a distinct methodological contribution to the time-use studies. Furthermore, Lee’s paper restrict itself to adults over the age of 65, while we focus on the working population. There is also no modelling of wellbeing nor focus on pandemic-related effects in Lee (2021). Please find the added section in our Literature Review below:

*“Lee (2021) focused on older adults over the age of 65 in the Korean Time Use Survey. Latent Class Analysis was used to identify four activity profiles across two waves in 2014 and 2019. The author aggregates daily activities into eight domains and summed up the time spent in each of these domains. The resulting eight continuous time measurements are then transformed into three-tier ordinal variables, which are used as the basis of classification. This approach focuses on the duration of activities, but ignores the timing and hence sequence of activities. As such, the study is not able to distinguish temporal patterns and sequences in its latent class assignments.”*

5. Looking at the number of total observations for each class (Appendix 5), I feel that the authors end up with too many classes and in some cases too few observations for each class. This study would probably benefit from other measures of model of fit and fewer classes or even a different method. These combined can provide to the analysis higher statistical power and more robust results.

Response: Thank you for this comment. Given the lived experience of significant lifestyle changes during the pandemic, it is a key aim of our paper to understand the transition from conventional commuting to more flexible home or hybrid working. Our conceptualisation of working flexibility features interconnected ‘spatial’ (commuting/home/hybrid working) and ‘temporal’ (early start/dispersed/nighttime etc.) dimensions. The combination of these two key dimensions enables a much more nuanced identification of lifestyles. While the number of total classes is high, it is clear from the visualisation that they represent distinct lifestyles that have not yet been explored in flexible working literature. We acknowledge that some classes have fewer samples, they still represent meaningful emerging lifestyles and deserve highlight in our paper. It is a difficult trade-off and we would hope that our nuanced lifestyle classification could be verified by future studies using data of larger sample size.

6. The authors claim that ExWB impact outside of usual working hours (before 6am and after 6pm) was consistently negative for all lifestyles. The choice of these thresholds seems quite arbitrary. Have other authors used such thresholds in the past? Why not for example before 9am and after 5pm? The authors should cite appropriately other authors that have used these thresholds or/and explain better and more the rationale of their choice.

Response: Thank you for raising this important point on the working hour thresholds. Our aim is to include the widest possible range of times that would be considered usual working times. Inherently there are subjective interpretations of when these thresholds should be. Our identification of the 6am-6pm window is data-driven - we see a consistent difference between the periods within and outside the 6am-6pm window across the ten lifestyles identified. While conventionally the 8am/9am-5pm worktime regimes are ingrained in many professional occupations, we take a wider range, as informed by the empirical data, in order to capture the temporal flexibility discussed in our paper.

7. I have concerns about the direction of causality between flexible/remote working and well-being. Some of the data were collected during the pandemic when mental health pressures were exceptionally high. Specifically, I am wondering whether flexible/remote working leads to changes in well-being, or if individuals with better (or worse) well-being are more inclined to work less (or more) flexibly/remotely and travel less (or more). Given the potential for reverse causality, it would be beneficial for this study to include a discussion on the direction of causality between flexible/remote working and ExWB.

While the authors do acknowledge some of the limitations of their study, they do not address potential issues of reverse causality or/and self-selection. So, it is strongly recommended that the authors explore alternative explanations for the observed associations, such as the potential for individuals with better (or worse) well-being to self-select into more (or less) flexible working arrangements.

Response: Thank you for raising the concern regarding causality in our analysis. A similar concern was also raised by the editor. We recognise that our study is focused on associative, rather than causal relationships between time use and wellbeing. This is due to the lack of longitudinal data and the absence of any suitable intervention (treatment) in our data. We have thus followed the suggestions of the editor and removed wording that allude to causality such as “impacts” from applicable places in our manuscript.

While we agree there are possible instances of circular causality, as our paper is only able to quantify associative relationship, the direction of causality is outside the scope of our consideration. However, if we are able to work with high quality longitudinal data in the future, the potential concerns of circular causality and self-selection would be extremely interesting to our subsequent research. We added the following clarification in the Limitations section of our manuscript:

*“Due to the non-interventional data, our study is not able to establish causal relationships – all findings are hence associative. Important issues such as the direction of causation and potentially circular causality cannot be explicitly modelled without longitudinal (and interventional) data. Using cross-sectional data, the possibility of self-selection bias cannot be ruled out. It is possible that workers with higher (lower) well-being are more inclined to work from home (office), and the ExWB difference arises not from the lifestyle choice of particular spatial or temporal working arrangements. However, the two-dimensional flexibility argument posits that if workers are able to self-select where and when they work, it could be a mechanism to avoid detriments to ExWB. Associative findings from our studies offer important insights and a promising avenue for future causal investigations.”*

8. “In this paper, the ExWB measure was transformed into a binary variable with enjoyment six and above defined as high ExWB, five and below defined as average-to-low ExWB.” The authors should explain the rationale of this choice. Have other authors used the same/similar definition? The authors should provide the specific wording/questions used from the time-survey(s) to construct their ExWB measure. Have authors tried alternative definitions? It would make sense for example to have a binary variable equal to 1 for enjoyment levels (5-7), and 0 otherwise, especially when the mean is a little bit above 5.

Response: Thank you for this very relevant question regarding the creation of the binary ExWB variable. Our primary consideration is the distribution of the ordinal scores please see details in the table below. In the pre-COVID data set, if the binary division is set per the reviewer’s suggestion at 5, then low ExWB (1-4) would only account for 25.77% of the samples. In the during COVID data set, low ExWB (1-4) would only account for 33.76% of the samples. This would make the binary classification less representative. As such, we approached the binary threshold in a data-driven way and set it roughly at the mid-point that splits the sample between 5 and below & 6 and above on the ordinal scale. The table below is appended to the manuscript as *S4 Appendix: ExWB descriptive statistics*.

|  | Pre-COVID | | | During COVID | | |
| --- | --- | --- | --- | --- | --- | --- |
| Level of Enjoyment | Frequency | Percentage | Cumulative | Frequency | Percentage | Cumulative |
| 1 | 2,396 | 1.53% | 1.53% | 2,617 | 1.53% | 1.53% |
| 2 | 4,649 | 2.96% | 4.49% | 3,303 | 1.93% | 3.46% |
| 3 | 11,103 | 7.08% | 11.57% | 11,087 | 6.49% | 9.95% |
| 4 | 22,280 | 14.20% | 25.77% | 40,692 | 23.81% | 33.76% |
| 5 | 35,588 | 22.68% | 48.45% | 40,134 | 23.48% | 57.24% |
| 6 | 37,149 | 23.68% | 72.13% | 29,916 | 17.50% | 74.24% |
| 7 | 43,718 | 27.87% | 100.00% | 43,181 | 25.26% | 100.00% |

9. BAME stands for Black, Asian, and Minority Ethnic groups, not Black, Asian, Middle Eastern groups as stated. Also, for robustness authors can think BME groups (Black and Minority Ethnic) only.

Response: We have amended the manuscript accordingly. Quoted below for your reference:

*“BAME (Black, Asian, Minority Ethnic) groups experienced greater distress in the first lockdown.”*

10. It is not clear why authors have used only UKTUS 2015, and the first online wave collected in 2016 to capture the pre-pandemic time-use. The authors should provide more information about any non-availability of data. For example, is this non-availability because the survey is conducted every 5 years? Aren’t data for other months from 2020 or 2021 available? In the case that authors have purposefully selected to use data for specific months they should justify adequately this selection.

Response: Thank you for your question regarding data availability. We used all available UKTUS data which were released at the point of submitting the original manuscript. The main UKTUS survey is conducted every five years. The 2016 data and three waves collected during COVID-19 utilised a new online surveying method enabling the higher sampling frequency. We have not purposefully selected data in any way, and we can confirm all available UKTUS data sets released before our original submission are included without gap or omission. We added the following clarification in the manuscript:

*“Data was obtained from the UK Data Service, all released data waves were included in our analysis without pre-selection or omission.”*

11. What is full employment? Full-time employment? If this is the case, is there any reason why part-time workers were excluded from the analysis? If specific groups of workers were excluded, then the possibility of sample selection bias arises. Women, for example, tend to work part-time to a greater extent than men, which could potentially pose a challenge to the study's validity. What about self-employed? Are they considered as home workers? Have self-employed been included or excluded?

Response: Thank you for raising this point for clarification. Full employment is used interchangeably as full-time employment in our analysis. We updated the manuscript to consistently use *“full-time employment”* to avoid confusion. We added the following to the Limitations section of the manuscript to clarify:

*“Our study sample only include those in full-time employment, as our aim is to identify latent lifestyles amongst a comparable pool of full-time (self-reported) workers. Including part-time workers would potentially confound the identification and interpretation of the latent classes. We identified groups of workers who are in full-time employment but enjoy greater spatial and temporal flexibility than others. Had self-declared part-time employees been included, we would not be able to separate these nuanced lifestyles amongst full-time workers. Self-employed workers are also excluded, as their flexibility to greater extents are self-determined. This is particularly relevant for our policy implications, as we argue against blanket “back-to-workplace” policies – these are most applicable to those employed on a full-time basis rather than the self-employed workers.”*

12. “The longitudinal dimension is enabled by combining population-representative, repeated cross-sectional time use data gathered during the pandemic (2020) with the pre-pandemic UK Time Use Survey (UKTUS) in 2015. I am not clear about the longitudinal dimension of the data. My impression has been that the data is cross-sectional. Authors should provide more details about the longitudinal dimension of the data, if there is any such a dimension.

Response: Thank you for this comment. We can confirm that the data is cross-sectional, the reviewer is correct in noting there is a lack of longitudinal dimension in our data, it is further discussed as a key limitation of our data. Please find the following relevant section in our manuscript:

*“Furthermore, the data from the UKTUS are population-representative but nevertheless cross-sectional. Higher quality longitudinal data, such as a cohort study would enable modelling analysis of causal relationship between lifestyles and ExWB.”*

13. According to Appendix 3, the distribution is left-skewed (skewness is negative); not right skewed as stated.

Response: Thank you for this important correction, we have changed the manuscript to left-skewed. As quoted below in the updated manuscript:

*“Our descriptive analysis found that the distribution of ordinal responses was heavily left-skewed, where the proportion of the top two scores (i.e., 6 – “Enjoyed” and 7 – “Enjoyed very much”) accounting for roughly half of overall responses (see distribution histogram in S3 Appendix and descriptive statistics of the distribution in S4 Appendix).”*

14. To motivate better their contribution and the importance of their findings, authors should think to provide literature and more evidence of how spatial and temporal flexibility looked like before and during the pandemic.

Response: Thank you for this helpful comment. Temporal flexibility has been largely overlooked in existing literature, the conceptualisation and quantification using TUS is an original contribution of this paper. We have added the following to the manuscript to provide more evidence on flexible working:

*“A systematic review on the impact of remote work on productivity found stark contrast where before the pandemic 79% studies showed positive effects of remote work, that figure dropped to 23% during the pandemic (Hackney et al., 2022). Preliminary evidence show that remote workers experience more anxiety around job security (Fosslien & Gottlieb-Cohen, 2023), and are more prone to loneliness (Miyake et al., 2022). Cell-phone-based mobility data reveal trips to the CBD stabilising at 60% of pre-pandemic levels, suggesting remote workers will make up a significant portion of the future workforce (Monte et al., 2023).”*

15. In order to enhance the readability of the results, it is recommended to include the initials for lifestyles, which are reported in Table 2, in parentheses in Appendix 5. This would facilitate the comprehension of the findings presented in the Appendix.

Response: Thank you for this useful suggestion. Appendix 5 (now *S6 Appendix*) has been updated to include the acronyms for each lifestyle.

**Responses to Reviewer #2**

This paper looks at the heterogeneous effects of wellbeing effects of the COVID-19 pandemic amongst the workforce in the UK. It specifically defines heterogeneous effects across lifestyles and work modes instead of the usual characteristics that have been used for this purpose in the literature (i.e. gender, ethnicity, income etc). Using the UKTUS the study found that experiential wellbeing is heterogeneous across workers lifestyle.

I agree with the authors on the importance of the study and the uniqueness of the time use data they have used. I generally think the paper is well-written and makes an important contribution by showing that both spatial and temporal flexibility is related to wellbeing.
I think the authors can improve on the clarity of the paper, I don’t think it will be easy for another author to replicate their result based on the description. For example, the implementation of the normalized entropy is not clear to me at all from the description. What entropy formulae was used and how exactly was it implemented? Similarly, since Plos one is interdisciplinary it might be useful to explain a bit more how the latent class analysis works and what it seeks to achieve. Lastly, the statistical analysis section mentioned that the LCA was conducted using pooled samples from pre-and during COVID. Why was this done this way? Why not have separate analyses for the two periods given that the dynamics can be different? To what extent does this affect the conclusions i.e. is the result robust to the choice of using pooled sample?

Response: Thank you for the helpful comments and cogent suggestions. We have restructured the section on the conceptualisation of the flexibility measurement to improve the clarity as seen in the quoted section:

*“A two-dimensional conceptual framework for measuring working flexibility is proposed, aiming to explicate the heterogenous wellbeing impacts across different lifestyles of workers. The spatial dimension measures the worker’s ability to choose the location to engage in paid work on an intraday basis. Spatial flexibility is a normalised entropy (see key formulae specified in S6 Appendix) measure of where workers are located during the working hours. The key variable measured is the location of an individual between the first and last hour for paid work. All locations recorded within the period are included in the entropy calculation, regardless of activity type.*

*Temporal flexibility is a distinct but interrelated dimension of flexibility, which concerns the ability to choose when to conduct paid work on an intraday basis, which is linked to how they organise their non-work activities including travel. The temporal flexibility is quantified using a normalised entropy (see key formulae specified in S6 Appendix) measuring when breaks related to Personal and Leisure activities, e.g. lunch breaks and short strolls, take place during the working hours. The daily working hours is identified by the timestamp of the first and last episode of paid work. One will achieve high temporal flexibility if they engage in numerous or prolonged breaks during the working hours. A higher entropy would derive from numerous or prolonged enjoyable breaks, indicating greater ability to organise one’s time, hence greater temporal flexibility.”*

The formulae for the Shannon entropy and its normalisation are added to the manuscript as:

*“S6 Appendix: Key formulae for normalised entropy measure for spatio-temporal flexibility*

$$H=-\sum_{i=1}^{N} X_{i}{log}_{2} X_{i}$$

*Shannon entropy (H) is a measure of the uncertainty of the value taken on by our variable investigated.* $X_{i}$ *denotes the probability that the variable X would take on the value observe at* $X_{i}$*.*

*In the case of spatial flexibility, there are three possible workplaces: home, office, other.* $X_{i}$ *is the probability density function of the three workplaces over the working day for all individuals belonging to the same lifestyle. Shannon entropy is calculated for each lifestyle and normalised using the min-max feature scaling formula.*

*For temporal flexibility, there are also three possible activities: personal, leisure, other.* $X_{i}$ *is the probability density function of enjoyable breaks over the working day for all individuals belonging to the same lifestyle. Shannon entropy is calculated for each lifestyle and normalised using the min-max feature scaling formula.*

$$H^{'}= \frac{H-H_{min}}{H_{max}-H_{min}}$$

*Where* $H^{'}$ *is the normalised entropy that has a range from 0-1, where 0 represents the highest level of synchronicity and 1 represents highest level of flexibility in relativity to the latent lifestyles.”*

The following section is added to the manuscript to explain the latent class analysis (LCA) and our motivation for adopting it in our methodology:

*“LCA is a generalised structural equation model used to* *identify a latent (unobserved) variable based on a set of observed categorical variables (Sinha et al., 2021). It is used to find groups with similar features or to decipher distinct patterns in multivariate data. The goal is to simplify and visualise complex and high-dimensional data in a data-driven method. Identification of LCA classes is particularly useful in largescale observational studies and helps contextualise unobserved heterogeneity.”*

Lastly, we pooled the samples from before and during COVID for the LCA models to ensure consistent lifestyle classifications. The use of LCA serves two purposes, 1) identifying latent yet distinct lifestyles among full-time workers; and 2) revealing the aggregate transition between lifestyles (i.e. the dominant class switching from conventional commuters to emergent homeworkers). Without pooling the samples together, the LCA results will be very difficult to compare even if the final class numbers are similar between two separate models.

By leveraging more advanced techniques such as machine learning, we may be able to compare a significantly larger number of class sizes. Meaningful interpretation of the bifurcated LCA models for before and during period, or potentially separating each wave collected in COVID is possible using a neural network framework. This would be extremely interesting for our subsequent research.

Further, the description of Table 2 (for logistical regression) suggests that there are additional controls including the interaction between activity type and lifestyle. Mentioning the interaction term for example will create the impression that these interaction coefficients are important (especially if they were significant) but the table neither shows this result nor discuss them. A related point is that when a model includes interaction terms, I don’t think the main effects can be interpreted the same way one would have without interaction terms, which then makes not presenting the full model’s result problematic.

I think if these concerns can be addressed the paper makes an important contribution to the literature.

Response: The interaction terms are indeed included in our model. We agree with the reviewer that the full model’s results should be reported, we have thus included this in the supplemental material please see below for your reference:

“*S8 Appendix: Full logistical regression model results*

|  | | Before COVID-19 | | During COVID-19 | |
| --- | --- | --- | --- | --- | --- |
|  |  | Coef | P>z | Coef | P>z |
| Time Category | Personal | 0.54 | 0.00 | 0.98 | 0.00 |
|  | Non-paid Work | 0.34 | 0.00 | 1.03 | 0.00 |
|  | Leisure | 1.23 | 0.00 | 1.34 | 0.00 |
|  | Travel | -0.05 | 0.26 | 0.55 | 0.00 |
| Lifestyle | Emergent Homeworker (EH) | 0.21 | 0.02 | -0.15 | 0.00 |
|  | Highflying Homeworker (HH) | 0.84 | 0.00 | 0.12 | 0.01 |
|  | Domestic Home-worker (DH) | 0.26 | 0.05 | -0.12 | 0.11 |
|  | Long-distance Commuter (LC) | -0.24 | 0.00 | -0.17 | 0.00 |
|  | Half-day Commuter (HC) | 0.33 | 0.00 | 0.19 | 0.00 |
|  | Nightshift Commuter (NC) | 0.22 | 0.09 | -0.37 | 0.04 |
|  | Conventional Hybrid (CH) | -0.09 | 0.14 | 0.34 | 0.00 |
|  | Long-hour Hybrid (LH) | 0.16 | 0.10 | 0.39 | 0.00 |
|  | Mobile Hybrid (MH) | 0.15 | 0.39 | -0.39 | 0.00 |
| Sex | Female | 0.05 | 0.01 | -0.23 | 0.00 |
| Age Group | 25-34 | -0.15 | 0.00 | 0.13 | 0.00 |
|  | 35-44 | -0.04 | 0.34 | 0.01 | 0.83 |
|  | 45-54 | -0.14 | 0.00 | -0.18 | 0.00 |
|  | 55-64 | 0.03 | 0.49 | -0.18 | 0.00 |
| Education | GCSEs | -0.03 | 0.57 | 2.11 | 0.00 |
|  | A Levels | -0.17 | 0.00 | 2.30 | 0.00 |
|  | First degree/ undergrad | -0.18 | 0.00 | 1.96 | 0.00 |
|  | Higher degree | -0.36 | 0.00 | 2.09 | 0.00 |
| Occupation | Skilled manual worker | -0.03 | 0.27 | 0.29 | 0.00 |
|  | Clerical / administrative | -0.11 | 0.01 | -0.19 | 0.00 |
|  | Supervisory/ junior managerial | -0.09 | 0.02 | -0.08 | 0.03 |
|  | Intermediate managerial | -0.09 | 0.01 | -0.10 | 0.01 |
|  | Higher managerial | -0.27 | 0.00 | -0.02 | 0.67 |
| Income | £1,000-1,999 | 0.27 | 0.00 | -0.53 | 0.00 |
|  | £2,000-2,999 | 0.12 | 0.00 | -0.40 | 0.00 |
|  | £3,000-3,999 | 0.02 | 0.54 | -0.55 | 0.00 |
|  | £4,000-5,000 | -0.18 | 0.00 | -0.60 | 0.00 |
|  | More than £5,000 | -0.13 | 0.00 | -0.26 | 0.00 |
| Marital Status | Married/living with partner | 0.16 | 0.00 | 0.30 | 0.00 |
|  | Divorced/widowed | 0.17 | 0.00 | 0.11 | 0.00 |
| Region | Yorkshire & Humberside | 0.12 | 0.01 | -0.32 | 0.00 |
|  | East Midlands | 0.05 | 0.23 | -0.75 | 0.00 |
|  | East Anglia | 0.17 | 0.00 | -0.25 | 0.00 |
|  | South East | -0.08 | 0.04 | -0.51 | 0.00 |
|  | South West | 0.07 | 0.14 | -0.76 | 0.00 |
|  | West Midlands | 0.26 | 0.00 | -0.62 | 0.00 |
|  | North West | 0.12 | 0.01 | -0.17 | 0.00 |
|  | Scotland | 0.48 | 0.00 | -1.36 | 0.00 |
|  | Wales | 0.07 | 0.21 | -0.49 | 0.00 |
|  | Northern Ireland | 0.44 | 0.00 | -0.39 | 0.00 |
|  | North East | 0.53 | 0.00 | -0.34 | 0.00 |
| TimeCategory#Lifestyle | Personal # EH | 0.18 | 0.18 | -0.16 | 0.01 |
|  | Personal # HH | -0.69 | 0.00 | -0.40 | 0.00 |
|  | Personal # DH | 0.06 | 0.72 | 0.18 | 0.06 |
|  | Personal # LC | -0.02 | 0.85 | -0.19 | 0.06 |
|  | Personal # HC | -0.13 | 0.12 | -0.21 | 0.05 |
|  | Personal # NC | 0.33 | 0.02 | 0.40 | 0.04 |
|  | Personal # CH | 0.03 | 0.76 | -0.44 | 0.00 |
|  | Personal # LH | -0.33 | 0.05 | -0.22 | 0.09 |
|  | Personal # MH | 0.08 | 0.71 | -0.24 | 0.10 |
|  | NPW # EH | -0.07 | 0.62 | -0.56 | 0.00 |
|  | NPW # HH | -0.68 | 0.01 | -2.24 | 0.00 |
|  | NPW # DH | -0.29 | 0.06 | -0.73 | 0.00 |
|  | NPW # LC | -0.11 | 0.32 | -0.36 | 0.09 |
|  | NPW # HC | -0.23 | 0.01 | 0.74 | 0.00 |
|  | NPW # NC | 0.04 | 0.79 | 0.15 | 0.48 |
|  | NPW # CH | 0.07 | 0.55 | -0.61 | 0.00 |
|  | NPW # LH | 0.24 | 0.29 | -0.50 | 0.22 |
|  | NPW # MH | -0.19 | 0.35 | -0.26 | 0.17 |
|  | Leisure # EH | 0.06 | 0.73 | -0.29 | 0.00 |
|  | Leisure # HH | -1.83 | 0.00 | -0.37 | 0.02 |
|  | Leisure # DH | -0.37 | 0.03 | -0.24 | 0.04 |
|  | Leisure # LC | -0.11 | 0.38 | -1.11 | 0.00 |
|  | Leisure # HC | -0.29 | 0.01 | -0.22 | 0.10 |
|  | Leisure # NC | 0.04 | 0.79 | 0.34 | 0.10 |
|  | Leisure # CH | -0.11 | 0.45 | -0.50 | 0.00 |
|  | Leisure # LH | 0.28 | 0.42 | -0.81 | 0.00 |
|  | Leisure # MH | -0.16 | 0.47 | -0.09 | 0.59 |
|  | Transport # EH | -0.51 | 0.01 | 0.05 | 0.66 |
|  | Transport # HH | -0.35 | 0.35 | 0.39 | 0.04 |
|  | Transport # DH | 0.01 | 0.97 | 0.01 | 0.93 |
|  | Transport # LC | 0.15 | 0.12 | 0.58 | 0.00 |
|  | Transport # HC | -0.03 | 0.74 | 0.08 | 0.61 |
|  | Transport # NC | 0.32 | 0.04 | 0.48 | 0.03 |
|  | Transport # CH | 0.07 | 0.53 | -0.02 | 0.89 |
|  | Transport # LH | -0.03 | 0.88 | 0.86 | 0.00 |
|  | Transport # MH | -0.08 | 0.73 | -0.11 | 0.57 |
