# Supplementary material for: Remote working and experiential wellbeing: A latent lifestyle perspective using UK time use survey before and during COVID-19
Source: PLoS One. 2024 Jul 25;19(7):e0305096. doi: 10.1371/journal.pone.0305096 (PMC11288641; doi:10.1371/journal.pone.0305096)
Supplement: Attachment — Submitted filename: Response to reviewers.docx [file pone.0305096.s011.docx]

**Comments to the Author**

1. If the authors have adequately addressed your comments raised in a previous round of review and you feel that this manuscript is now acceptable for publication, you may indicate that here to bypass the “Comments to the Author” section, enter your conflict of interest statement in the “Confidential to Editor” section, and submit your "Accept" recommendation.

Reviewer #1: (No Response)

2. Is the manuscript technically sound, and do the data support the conclusions?

The manuscript must describe a technically sound piece of scientific research with data that supports the conclusions. Experiments must have been conducted rigorously, with appropriate controls, replication, and sample sizes. The conclusions must be drawn appropriately based on the data presented.

Reviewer #1: Yes

3. Has the statistical analysis been performed appropriately and rigorously?

Reviewer #1: Yes

4. Have the authors made all data underlying the findings in their manuscript fully available?

The [PLOS Data policy](https://eur03.safelinks.protection.outlook.com/?url=http%3A%2F%2Fwww.plosone.org%2Fstatic%2Fpolicies.action%23sharing&data=05%7C02%7Cjc2205%40cam.ac.uk%7C8a1b78e23ff7451ca1fe08dc2286ecf4%7C49a50445bdfa4b79ade3547b4f3986e9%7C1%7C0%7C638423210101158403%7CUnknown%7CTWFpbGZsb3d8eyJWIjoiMC4wLjAwMDAiLCJQIjoiV2luMzIiLCJBTiI6Ik1haWwiLCJXVCI6Mn0%3D%7C0%7C%7C%7C&sdata=taXfC7b6wflJiZshv6r4np5kOjxHs5nhYHq%2FeDcA3Lo%3D&reserved=0) requires authors to make all data underlying the findings described in their manuscript fully available without restriction, with rare exception (please refer to the Data Availability Statement in the manuscript PDF file). The data should be provided as part of the manuscript or its supporting information, or deposited to a public repository. For example, in addition to summary statistics, the data points behind means, medians and variance measures should be available. If there are restrictions on publicly sharing data—e.g. participant privacy or use of data from a third party—those must be specified.

Reviewer #1: Yes

5. Is the manuscript presented in an intelligible fashion and written in standard English?

PLOS ONE does not copyedit accepted manuscripts, so the language in submitted articles must be clear, correct, and unambiguous. Any typographical or grammatical errors should be corrected at revision, so please note any specific errors here.

Reviewer #1: Yes

6. Review Comments to the Author

Please use the space provided to explain your answers to the questions above. You may also include additional comments for the author, including concerns about dual publication, research ethics, or publication ethics. (Please upload your review as an attachment if it exceeds 20,000 characters)

Reviewer #1: Referee's Report
Manuscript PONE-D-23-04776R1
'Remote working and experiential wellbeing: A latent lifestyle perspective using UK Time Use Survey before and during COVID-19'

I would like to express my appreciation to the authors for their efforts in addressing the feedback provided. You can find a list of comments below that highlight specific recommendations for further enhancing the paper's potential to offer valuable insights to the field of well-being and remote work. While recognizing the inherent complexities of this research and the limitations posed by available data, I believe that a more detailed explanation of these aspects would significantly strengthen the paper’s overall quality.

With regards to authors’ response to my previous concerns:

1. The length of the abstract is now reduced. However, the authors refer to the time range of 6 am to 6 pm as 'usual working hours' without providing a reference for this threshold later in the paper. Since there is no provided reference for this characterisation (according to authors’ response in comment 6), it would be more accurate to avoid such a characterisation in the abstract and throughout the paper.

Response: Thank you for this important reminder. We can confirm that all instances of our characterisation of ‘usual working hour’ have been removed from the abstract and throughout the paper. Instead, we conceptualise and differentiate between “daytime activities window (between 6am and 6pm)” and “evening activities window (before 6am and after 6pm)” to clearly bifurcate the 24-hour day into two 12-hour observational windows which are important for our subsequent analysis. The following passage has been added to the manuscript in the Results Section with additional references provided:

“To better understand the heterogeneity observed across the time of the day, we bifurcate the diary day into two 12-hour windows: for daytime activities (between 6am and 6pm) and for evening activities (before 6am and after 6pm). The division recognises the stratifying influence of institutional-organisational time (Scott, 1987). As we use full employment as an inclusion criterium, within-sample must account for collective time schedules – such as those commonly prescribed by the employer during the daytime activities (Flood et al., 2018). We focus primarily on the daytime activities window (between 6am and 6pm) as significant heterogeneity is observed across activities and lifestyles during these hours. In addition to the empirical observation that informed the selection of temporal boundaries for daytime and evening, this window maximises the diversity and richness of daytime activities captured by the granular intra-day temporal data. It expands on the conventional paid work window (such as 9am-5pm), which excludes important activities such as travel and non-paid work which reflect the distinct lifestyles we identified. The 6am and 6pm temporal boundaries result in two comparable observational windows which refines our focus to improve understanding of ExWB and flexible working.”

2. I appreciate the authors' response and the rationale provided for not including parental status and household composition as covariates in their specification. However, I would like to emphasize the significance of the endogeneity concern in this context. Endogeneity is a bias that can substantially affect the credibility of the study’s findings. As such, it should not be considered 'out of the scope' of the paper. While the authors have stated their reasons for not including these variables, it is crucial that the limitations section of the manuscript more explicitly captures this issue. Specifically, the authors should rephrase their limitation statement to emphasise the potential endogeneity of parental status and household composition in relation to ExWB and remote work and its relevance to their work. They should highlight the complexity of the relationships involved and the need for further research.

Response: Thank you to the reviewer for raising the concern regarding endogeneity arising from not controlling for parental status and household composition. We have followed your instruction and revised the limitation statement accordingly. Please see the revised paragraph in the Limitation Section, quoted below:

“The logistical regression model does not include parental status and household composition as covariates. The primary consideration for excluding household composition (including parental status) is a significant non-response issue in the original data where 20% of study samples did not provide the number of children. Inclusion of the covariate would significantly reduce the samples size, leading to identification issues. However, this exclusion may give rise to endogeneity in the logistical regression model. Firstly, childcare and eldercare hours are highly endogenous to the enjoyment of non-paid work, comparing the enjoyment levels during non-paid work activities without explicitly controlling for care duty may risk confounding the coefficients estimated. Secondly, household composition and parental status are important sociodemographic characteristics, the exclusion may lead to incompleteness in the model parameters. Thirdly, without controlling for differences in household composition and parental status weakens the generalisability when comparing ExWB changes before and after the pandemic. Further research is needed to expand the observational unit to the household rather than at the individual level to address the complexities and better address potential endogeneity.”

3. Could the authors provide a more detailed explanation or illustration of how the latent class models are employed to capture the heterogeneity in ExWB changes among different worker lifestyles? It would be beneficial to understand the specific mechanisms or statistical techniques used within these models that enable a better control of heterogeneity. A more comprehensive description in this regard would greatly enhance the clarity of the methodology and its relevance to the issue of heterogeneous effects, which is a critical aspect of this study.

Response: Thank you for raising the need for further explaining the LCA model specifications. While the key considerations for model choices are summarised at the start of the Statistical Analysis Section:

“Latent class analysis (LCA) is used to identify nuanced lifestyles within each working mode group (homeworkers, commuters, and hybrid workers). LCA is a generalised structural equation model used to identify a latent (unobserved) categorical variable based on a set of observed categorical variables (Sinha et al., 2021). It is used to find groups with similar features or to decipher distinct patterns in multivariate data through maximizing cross-group variance and minimising within-group variance. The goal is to simplify and visualise complex and high-dimensional data in an unsupervised manner. Identification of LCA classes is particularly useful in largescale observational studies and helps contextualise unobserved heterogeneity. In particular, the relatively homogenous groups identified as a result of the LCA models have distinctive spatial and temporal features representing interpretable lifestyles. The identification of lifestyle delineates heterogenous subgroups of the sample that would have otherwise aggregated as a homogenous sample, mitigating the risk of inadvertently averaging out important heterogeneities in these unidentified latent subgroups. For key formulae and technical specifications see *S5 Appendix*.”

Furthermore, we agree with the reviewer that given the importance of the LCA methodological choice, the technique should be explained in greater detail. We thus added the key equations and parameters in the Supplemental Materials:

*“S5 Appendix: Key formulae for LCA*

$$P_{jkt}= P_{t}^{X}P_{jt}^{AX}P_{kt}^{BX}$$

(Equation 1)

*X* is identifiable as a latent variable with $T=1\ldots t$ classes so that $P_{t}^{X}$is the probability of being in latent class *t*. $P_{jt}^{AX}$ is the probability of being in category *j* of variable A conditional on being in latent class *t*. $P_{kt}^{BX}$is the probability of being in category *k* of variable B conditional on being in latent class *t*. $P_{jkt}$ is the joint probability of being in category *j*, category *k,* and class *t.*

The responses of individual *i* on variables A and B are denoted each as $Y_{ijk}$, which is represented in the following section as a vector $Y_{i}$, such that the latent class model is defined as:

$$P\left( Y_{i} \right)=\sum_{t=1}^{T} P(X=t)P(Y_{i}|X=t)$$

(Equation 2)

Where $P(X=t)$ denote the class proportions. Applying a simple Bayesian formula to obtain the probability of belonging to latent class *t* given the observed response vector $Y_{i}$, the posterior membership probability is obtained from (Weller et al., 2020):

$$P\left( X=t | Y_{i} \right)=\frac{P(X=t)P(Y_{i}|X=t)}{P\left( Y_{i} \right)}$$

(Equation 3)

Further defining the assigned latent class membership of individual *i* as $W_{i}$*.* Adopting a proportional classification, which yields a soft partitioning with boundary *s* for assigning individuals to latent class *t* denoted as $W_{it}=P\left( W_{i}=t | Y_{i} \right)=P\left( X=t | Y_{i} \right).$ Allowing the classification error to be defined in terms of probability of the estimated value conditional on the true value:

$$P\left( W=s | X=t \right)=\frac{\sum_{i=1}^{N} P\left( X=t | Y_{i} \right)P\left( W=s | Y_{i} \right)}{P(X=t)}=\frac{\sum_{i=1}^{N} W_{it}W_{is}}{P(X=t)}$$

(Equation 4)

24 hourly categorical variables indicating activity type were used as the observed categorical variables in the models, together with categorical socioeconomic covariates including income quantile, education attainment, and occupation classification. Following the three-step model (Bolck et al., 2004), incorporating covariate with response vector $Z_{i}$ into the latent class model allows the covariates effects to be simultaneously estimated with the parameters defining the class-specific item distributions such that:

$$P\left( W=s|Z_{i} \right)=\sum_{t=1}^{T} P(X=t|Z_{i})P\left( W=s | X=t \right)$$

(Equation 5)

A multinomial logistical regression is used to parametrise $P(X=t|Z_{i})$. Using a maximum log-likelihood function, the model parameters above are estimated with:

$$LogL=\sum_{i=1}^{N} \log P\left( Y_{i} | Z_{i} \right)=\sum_{i=1}^{N} \log\sum_{t=1}^{T} P(X=t|Z_{i})P\left( W=s | X=t \right)$$

(Equation 6)

To ensure the global rather than local maximum is identified in the maximum likelihood estimation, Newton-Raphson stepping mechanism guarantees convergence if the initial estimate $x_{0}$ are close enough to the true estimates (Smith, 1998), as shown in the formula:

$$x_{n+1}=x_{n}-\frac{f(x_{n})}{f'(x_{n})}$$

(Equation 7)

To narrow the initial estimates to aid model convergence under Newton method, we apply a two-step integration process in the generalised linear mixed model used for LCA: Laplacian approximation was used in the first round of integration, the resulting matrix was then used as the starting value for the more accurate mean-variance adaptive Gauss-Hermite quadrature integration method (Rabe-Hesketh et al., 2002):

$$\int_{-\infty}^{\infty} L(\beta_{0}, \beta_{1};Y_{i}|X=t)P(X=t|Z_{i})dx$$

(Equation 8)

$$LogL\left( \beta_{0}, \beta_{1};Y_{i} | X=t \right)=Y_{i}\left( \beta_{0}+\beta_{1}x \right)-\log(1+e^{\beta_{0}+\beta_{1}x})$$

(Equation 9)

One to six class models are iteratively attempted, the final class size within each working mode was determined using the Akaike's information criterion (AIC) and Bayesian information criterion (BIC) values, average latent class probabilities, and substantive interpretation of the classes identified:

$$AIC=-2\log L\left( \hat{\theta} \right)+2k$$

(Equation 10)

$$BIC=-2\log L\left( \hat{\theta} \right)+k\log n$$

(Equation 11)

Where $\theta$ is the vector of model parameters and $k$ is the number of estimated parameters. $L\left( \hat{\theta} \right)$ is the candidate model when evaluated at the maximum likelihood estimate of $\theta$. LCA was conducted using pooled samples from both pre- and during COVID-19. After LCA, the samples were then separated into pre- and during COVID-19 groups according to the survey date.”

*Note: please kindly note there was no Comment 4 from the reviewer in the Decision Letter, possibly due to numbering error (i.e. Comment 3 above immediate preceded Comment 5 below). We thus answer Comment 5 below without re-numbering the comments for consistency.

5. The authors recognize that some classes have fewer samples, which can affect statistical power and the robustness of the results. While they argue that these smaller classes still represent meaningful emerging lifestyles, the issue of statistical power remains a valid concern and should be properly discussed in either the conclusion or limitations section.

Response: Thank the reviewer for raising the concern regarding the uneven sizes of latent classes. We acknowledge this limitation and have added the following discussion to the Limitation Section:

“A minority of lifestyles identified from LCA models have relatively small class sizes, in particular two of the hybrid lifestyles during the pandemic (LH and MH have 30 and 36 individuals respectively). Compared to lifestyles with larger class size, the ExWB averages produced may be subject to the effects of outliers. While the logistical regression produced highly statistically significant estimates for the smaller LH and MH lifestyles during the pandemic (>99% significance), it does not rule out possible limitation affecting the external validity of these estimates due to smaller sample sizes. This limitation resulted from a confluence of a minority of workers engaging in flexible working lifestyles such as hybrid lifestyles, as well as the data restriction of fewer individuals in the during-COVID dataset. As hybrid working becomes more prevalent and more post-pandemic data is gathered, it would be useful to verify the external validity of the findings for the smaller LCA classes of hybrid workers in future studies.”

6. While I appreciate the authors' response and the rationale provided for choosing the working hour thresholds of 6 am to 6 pm, it is essential to enhance the transparency of this choice for the benefit of readers. I recommend adding a more detailed explanation for this choice in the measurements section of the manuscript.

Response: Thank you for the cogent comment. We agree and have revised the characterisation following the reviewer’s Comment 1. We appreciate the reviewer’s concern regarding the need to signpost this methodological choice earlier in the manuscript, we added the following passage to the Measurement Section:

“Recognising the rigidity of certain working arrangement (e.g. the eight-hour working day that has largely persisted since the industrial era) and the need to capture lifestyle differences before and during COVID-19, the dairy day for each respondent is bifurcated into a “daytime activities window” (between 6am and 6pm) and a “evening activities window” (before 6am and after 6pm). The 12 consecutive categorical hourly variables thus fall into each observational window in a mutually exclusive manner, with 6am and 6pm as the boundaries. The rationale for these temporal boundaries is based on empirical observations (see details in the Results section).”

7. I appreciate the authors' response regarding the concern of causality in the analysis and their efforts to eliminate language implying causality from the manuscript. To ensure consistency throughout the paper and align with the authors' intent to focus on associative relationships, I recommend a careful proofreading of the entire manuscript. This would help identify and appropriately adapt any remaining instances of language suggesting causality. Consistency in terminology will enhance the clarity and accuracy of the paper's purpose.

Response: Thank the reviewer for raising the importance of consistency regarding the use of associative relationships, we confirm that this issue has now been addressed throughout the manuscript. In particular, we confirm that any references to causal language such as “impacts” or “effects” have been replaced with “relationship between” or “association”.

*Note to editor regarding changes in the reference list, the following two sources have been added during the revision process:

Flood, S.M., Hill, R., Genadek, K.R., 2018. Daily Temporal Pathways: A Latent Class Approach to Time Diary Data. Soc Indic Res 135, 117–142. <https://doi.org/10.1007/s11205-016-1469-0>

Scott, W.R., 1987. The Adolescence of Institutional Theory. Administrative Science Quarterly 32, 493–511. <https://doi.org/10.2307/2392880>

And a duplicate citation found in the reference list has been removed:

Holmes, E.A., O’Connor, R.C., Perry, V.H., Tracey, I., Wessely, S., Arseneault, L., Ballard, C., Christensen, H., Cohen Silver, R., Everall, I., Ford, T., John, A., Kabir, T., King, K., Madan, I., Michie, S., Przybylski, A.K.,

*Note to editor regarding changes in data availability statement after receiving helpful advice from the editorial team communicated to the authors on March 20, 2024. While the source datasets are publicly available and hosted on the UK Dataset, we clarified the previous Code Availability section and added a dedicated Data Availability Statement section:

## “Data availability statement

Data cannot be shared publicly because it is owned by the UK Data Service. Data are available directly from the UK Data Service for researchers who meet the criteria for access to confidential data.

The data underlying the results presented in the study are directly accessible via the DOIs below:

http://doi.org/10.5255/UKDA-SN-8128-1

http://doi.org/10.5255/UKDA-SN-8741-3

After downloading the source datasets from UK Data Service, follow instructions included in the STATA DO file for reproduction of results. All codes have been made publicly available via the project GitHub repository, and can be accessed via the URL below:

https://github.com/jerchenn/TimeUseWellbeing”

7. PLOS authors have the option to publish the peer review history of their article ([what does this mean?](https://eur03.safelinks.protection.outlook.com/?url=https%3A%2F%2Fjournals.plos.org%2Fplosone%2Fs%2Feditorial-and-peer-review-process%23loc-peer-review-history&data=05%7C02%7Cjc2205%40cam.ac.uk%7C8a1b78e23ff7451ca1fe08dc2286ecf4%7C49a50445bdfa4b79ade3547b4f3986e9%7C1%7C0%7C638423210101166131%7CUnknown%7CTWFpbGZsb3d8eyJWIjoiMC4wLjAwMDAiLCJQIjoiV2luMzIiLCJBTiI6Ik1haWwiLCJXVCI6Mn0%3D%7C0%7C%7C%7C&sdata=i5eMyEMVS0p1LzzrHG5nWyVVJqTgxZbZotBFGmz1sJE%3D&reserved=0)). If published, this will include your full peer review and any attached files.


If you choose “no”, your identity will remain anonymous but your review may still be made public.


**Do you want your identity to be public for this peer review?** For information about this choice, including consent withdrawal, please see our [Privacy Policy](https://eur03.safelinks.protection.outlook.com/?url=https%3A%2F%2Fwww.plos.org%2Fprivacy-policy&data=05%7C02%7Cjc2205%40cam.ac.uk%7C8a1b78e23ff7451ca1fe08dc2286ecf4%7C49a50445bdfa4b79ade3547b4f3986e9%7C1%7C0%7C638423210101172518%7CUnknown%7CTWFpbGZsb3d8eyJWIjoiMC4wLjAwMDAiLCJQIjoiV2luMzIiLCJBTiI6Ik1haWwiLCJXVCI6Mn0%3D%7C0%7C%7C%7C&sdata=QmK1EJkgpyzeNhv2rTF%2FRP%2BBRvsRzHkCHZASNs1rX7Y%3D&reserved=0).

Reviewer #1: No

[NOTE: If reviewer comments were submitted as an attachment file, they will be attached to this email and accessible via the submission site. Please log into your account, locate the manuscript record, and check for the action link "View Attachments". If this link does not appear, there are no attachment files.]

While revising your submission, please upload your figure files to the Preflight Analysis and Conversion Engine (PACE) digital diagnostic tool, [https://pacev2.apexcovantage.com/](https://eur03.safelinks.protection.outlook.com/?url=https%3A%2F%2Fpacev2.apexcovantage.com%2F&data=05%7C02%7Cjc2205%40cam.ac.uk%7C8a1b78e23ff7451ca1fe08dc2286ecf4%7C49a50445bdfa4b79ade3547b4f3986e9%7C1%7C0%7C638423210101178239%7CUnknown%7CTWFpbGZsb3d8eyJWIjoiMC4wLjAwMDAiLCJQIjoiV2luMzIiLCJBTiI6Ik1haWwiLCJXVCI6Mn0%3D%7C0%7C%7C%7C&sdata=bwstOJQcv8OGMS7wm93%2BjIghxNgA8DxTbB5gDdip7Ls%3D&reserved=0). PACE helps ensure that figures meet PLOS requirements. To use PACE, you must first register as a user. Registration is free. Then, login and navigate to the UPLOAD tab, where you will find detailed instructions on how to use the tool. If you encounter any issues or have any questions when using PACE, please email PLOS at [figures@plos.org](mailto:figures@plos.org). Please note that Supporting Information files do not need this step.
