# Supplementary material for: Remote working and experiential wellbeing: A latent lifestyle perspective using UK time use survey before and during COVID-19
Source: PLoS One. 2024 Jul 25;19(7):e0305096. doi: 10.1371/journal.pone.0305096 (PMC11288641; doi:10.1371/journal.pone.0305096)
Supplement: Attachment — Submitted filename: Response to reviewers.docx [file pone.0305096.s012.docx]

PONE-D-23-04776R2

Remote working and experiential wellbeing: A latent lifestyle perspective using UK Time Use Survey before and during COVID-19

PLOS ONE

Dear Dr. Wan,

Thank you for submitting your manuscript to PLOS ONE. After careful consideration, we feel that it has merit but does not fully meet PLOS ONE’s publication criteria as it currently stands. Therefore, we invite you to submit a revised version of the manuscript that addresses the points raised during the review process.

Please submit your revised manuscript by Jun 01 2024 11:59PM. If you will need more time than this to complete your revisions, please reply to this message or contact the journal office at [plosone@plos.org](mailto:plosone@plos.org). When you're ready to submit your revision, log on to <https://www.editorialmanager.com/pone/> and select the 'Submissions Needing Revision' folder to locate your manuscript file.

Please include the following items when submitting your revised manuscript:

- A rebuttal letter that responds to each point raised by the academic editor and reviewer(s). You should upload this letter as a separate file labeled 'Response to Reviewers'.
- A marked-up copy of your manuscript that highlights changes made to the original version. You should upload this as a separate file labeled 'Revised Manuscript with Track Changes'.
- An unmarked version of your revised paper without tracked changes. You should upload this as a separate file labeled 'Manuscript'.

If you would like to make changes to your financial disclosure, please include your updated statement in your cover letter. Guidelines for resubmitting your figure files are available below the reviewer comments at the end of this letter.

If applicable, we recommend that you deposit your laboratory protocols in protocols.io to enhance the reproducibility of your results. Protocols.io assigns your protocol its own identifier (DOI) so that it can be cited independently in the future. For instructions see: [https://journals.plos.org/plosone/s/submission-guidelines#loc-laboratory-protocols](https://eur03.safelinks.protection.outlook.com/?url=https%3A%2F%2Fjournals.plos.org%2Fplosone%2Fs%2Fsubmission-guidelines%23loc-laboratory-protocols&data=05%7C02%7Cjc2205%40cam.ac.uk%7C3d3a0e3b65cb44b487a308dc5eec9d8e%7C49a50445bdfa4b79ade3547b4f3986e9%7C1%7C0%7C638489617546932792%7CUnknown%7CTWFpbGZsb3d8eyJWIjoiMC4wLjAwMDAiLCJQIjoiV2luMzIiLCJBTiI6Ik1haWwiLCJXVCI6Mn0%3D%7C0%7C%7C%7C&sdata=M%2BFJ9%2BWbZBlez8NCCCtQhmBAw4i24%2BOIDvUxfzrGhG8%3D&reserved=0). Additionally, PLOS ONE offers an option for publishing peer-reviewed Lab Protocol articles, which describe protocols hosted on protocols.io. Read more information on sharing protocols at [https://plos.org/protocols?utm_medium=editorial-email&utm_source=authorletters&utm_campaign=protocols](https://eur03.safelinks.protection.outlook.com/?url=https%3A%2F%2Fplos.org%2Fprotocols%3Futm_medium%3Deditorial-email%26utm_source%3Dauthorletters%26utm_campaign%3Dprotocols&data=05%7C02%7Cjc2205%40cam.ac.uk%7C3d3a0e3b65cb44b487a308dc5eec9d8e%7C49a50445bdfa4b79ade3547b4f3986e9%7C1%7C0%7C638489617546952459%7CUnknown%7CTWFpbGZsb3d8eyJWIjoiMC4wLjAwMDAiLCJQIjoiV2luMzIiLCJBTiI6Ik1haWwiLCJXVCI6Mn0%3D%7C0%7C%7C%7C&sdata=9e64%2FAbSajP0Y%2F5vR73XCM7%2Fa4SFXeIc%2FdBfeBSjmkY%3D&reserved=0).

We look forward to receiving your revised manuscript.

Kind regards,

José Alberto Molina
Academic Editor
PLOS ONE

Journal Requirements:

Please review your reference list to ensure that it is complete and correct. If you have cited papers that have been retracted, please include the rationale for doing so in the manuscript text, or remove these references and replace them with relevant current references. Any changes to the reference list should be mentioned in the rebuttal letter that accompanies your revised manuscript. If you need to cite a retracted article, indicate the article’s retracted status in the References list and also include a citation and full reference for the retraction notice.

6. Review Comments to the Author

Please use the space provided to explain your answers to the questions above. You may also include additional comments for the author, including concerns about dual publication, research ethics, or publication ethics. (Please upload your review as an attachment if it exceeds 20,000 characters)

Reviewer #1: No further comments. There are still some minor grammatical errors and typos. For example, 'Limitation' section should be 'Limitations'. I recommend a full proofreading of the paper.

Response: Thank you to the reviewer for the reminder on grammatical accuracy. We confirm a full proofreading has been undertaken by both authors. We made the following changes in the proofreading process:

1. Section head “Limitation” was revised as “Limitations”;
2. In Abstract, “It was found that” was deleted for passive voice;
3. In Introduction, “and social scarring” was deleted for better readability;
4. In Introduction, comma added for readability: “… the lifestyle perspective and the temporal dimension combined are new, thus contribute to our understanding of the association between flexible/remote working and wellbeing.”;
5. In Literature Review, “as well” was removed: “…poses a large burden for the respondents” to improve readability;
6. In Literature Review, “taken place” was removed;
7. In Literature Review, the acronym “ONS” has been expanded to “Office for National Statistics”;
8. In Data and Methods, “endogenous” was removed and “mode” was changed to model as a correction for spelling in the sentence “Prior to the identification of lifestyles using the latent class models…”;
9. In Data and Methods, “they” was clarified as “workers”;
10. In Results, Table 1, “demographies” was changed to “samples”; “working from home” was changed to “homeworking”; “lockdown” was changed to “COVID-19”; “spends the most time” was changed to “spends the longest duration”;
11. In Results, “homeworker groups” was changed to “homeworking lifestyles”;
12. In Results, “each reflecting” was changed to “respectively for”;
13. In Results, “turn to negative” was changed to “turn negative”;
14. In Results, two instances of “effect” was changed to “changes” to avoid causal insinuation;
15. In Conclusion, “It was found that” was deleted for passive voice.

All changes are recorded in the revised manuscript using tracked changes.

Reviewer #3: The current version of the paper is quite well. Two final comments:

1 If the UKTUS is cross-sectional, and additional waves to the 2015 wave are “population-representative”, I wonder whether individuals' assessment of well-being at work might be affected by whether or not individuals remain employed. Let me explain my point of view. If many people lost their jobs, or are on flexible time schemes not working, during the lockdowns, individuals who remain employed during COVID may feel better off compared to similar individuals Pre-COVID, simply because in "bad times" they are still employed. This "possible" source of bias may occur regardless of lifestyle, and worker mode. The authors may find this hypothesis relevant.

Response: Thank you to the reviewer for the cogent comment regarding the possible upward bias in ExWB for those who remain employed during the COVID pandemic. The reviewer posed a valid and intriguing hypothesis – given the broader social context of mass layoffs or furlough schemes, it is possible that individuals who keep their employment receive a ExWB increase. We refer this possible ExWB increase for those remaining employed as ‘background effect' in the following discussion.

Firstly, our samples are restricted to full time workers in both UKTUS (2015) and the following waves. It is, however, not possible to track the change of employment status over time due to the cross-sectional nature of our samples. We observed highly heterogenous changes in ExWB (both in direction and magnitude) during the pandemic – particularly during hours with high concentrations of workers engaging in paid work. To investigate whether, and if so, how the ‘background effect’ would influence our findings, it is important to refine the hypothesis by considering whether the effect is uniform among those remained full-time employed. We will discuss two cases below.

If the background effect is uniform among our full-time worker samples, it may introduce a uniform bias (in terms of the lifestyle groups) to our logistic regression model estimates. However, the core finding on heterogenous changes in ExWB (both in direction and magnitude) remains valid, as the uniform bias would not alter the relative differences between lifestyles (the identification of which is independent of ExWB measures).

If the background effect is non-uniform among our samples, such effect, in theory, has already been accounted for in our empirical models as part of the observed heterogeneity. In other words, the possible existence of background effect would support our core finding, though likely to derive from a different source. We agree with the reviewer that it would be useful to investigate and isolate the various sources of the heterogenous effects on ExWB, but such a task would require a causal framework which falls out of the scope of the paper. We have made considerable efforts in the previous revisions to clarify that our study is not causal but based on association.

Based on the above analysis, we appreciate that the background effect as hypothesised by the reviewer helps us to gain a deeper understanding of the observed heterogenous effects on ExWB. The possible existence of the background effect does raise a potential issue of biased estimates in our logistic models, but overall it does not alter our core findings. On the contrary, it may corroborate our findings if the background effect itself was heterogenous across employed workers. We would like to investigate the possible linkage between broader labour market changes and ExWB in future studies. Accordingly, we added the following passage to our Limitations section to clarify the potential bias:

“The lack of longitudinal data limits our ability to control for employment change across waves, which we addressed by restricting samples to full-time workers at each data cross-section for comparability. On an aggregate level, the logistical regression model results cannot capture changes in the labour market and the associated background effects on ExWB. Specifically, the background effects may be linked to an upward bias in ExWB at the start of the pandemic, as our samples were fully employed despite the labour market contraction. If such background effects were present and uniformly impacted samples during COVID-19, the relative differences in ExWB across lifestyles would remain constant. On the other hand, non-uniform background effects may have contributed to, and help explain, the observed heterogeneity between lifestyles. In either case, background effects associated with labour market changes would not contradict our core findings. Longitudinal data would be required to identify the presence of such effects, and to delineate the sources of heterogeneity.”

2 In the section "Time use and ExWB during the evening activities window", first sentence. "ExWB decreased uniformly across all lifestyles within the evening activities". I don't quite understand the sentence. The later in the evening the less satisfied with activities or only when working? or is there some gradation in lifestyles? I may have misunderstood the process.

Response: Thank you for the comment. In the sentence highlighted by the reviewer, we are comparing the evening activities window pre- and during COVID-19, rather than the trend within the evening activities window itself (i.e. early evening versus later in the evening). Empirical results show a decline of ExWB in the evening activities window during COVID-19, compared against the pre-COVID level, and the reduction is uniform across all lifestyles. We do not observe a significant temporal gradation in ExWB within the evening activities across lifestyles. We have revised this sentence for better clarity. Please see the revision below:

“During COVID, ExWB declined for all lifestyles within the evening activities window when most people engaged in leisure or personal activities such as sleeping.”
